# The Single-Stranded RNA Bacteriophage Qβ Adapts Rapidly to High Temperatures: An Evolution Experiment

**DOI:** 10.3390/v12060638

**Published:** 2020-06-12

**Authors:** Md. Tanvir Hossain, Toma Yokono, Akiko Kashiwagi

**Affiliations:** 1 The United Graduate School of Agricultural Science, Iwate University, Morioka 020-8550, Japan; i80217010@hirosaki-u.ac.jp; 2 Faculty of Agriculture and Life Science, Hirosaki University, Hirosaki 036-8561, Japan; one.of.a.kind.12162127@gmail.com

**Keywords:** ssRNA phage Qβ, thermal adaptation, experimental evolution

## Abstract

Single-stranded (ss)RNA viruses are thought to evolve rapidly due to an inherently high mutation rate. However, it remains unclear how ssRNA viruses adapt to novel environments and/or how many and what types of substitutions are needed to facilitate this evolution. In this study, we followed the adaptation of the ssRNA bacteriophage Qβ using thermally adapted *Escherichia coli* as a host, which can efficiently grow at temperatures between 37.2 and 45.3 °C. This made it possible to evaluate Qβ adaptation to the highest known temperature that supports growth, 45.3 °C. We found that Qβ was capable of replication at this temperature; within 114 days (~1260 generations), we detected more than 34 novel point mutations in the genome of the thermally adapted Qβ population, representing 0.8% of the total Qβ genome. In addition, we returned the 45.3 °C-adapted Qβ populations to 37.2 °C and passaged them for 8 days (~124 generations). We found that the reverse-adapted Qβ population showed little to no decrease in fitness. These results indicate that Qβ can evolve in response to increasing temperatures in a short period of time with the accumulation of point mutations.

## 1. Introduction

Bacteriophages are ubiquitous viruses and are estimated to be among the most widely distributed and diverse entities in the biosphere [1]. In the global ecosystem, bacteria, which are the targets and hosts of bacteriophages, play important functions in modulating the nutrient and/or energy cycles [2]. In general, bacteriophages are highly specific for a given host; lytic bacteriophages kill the host cell to release their progeny. Therefore, preferential replication of a specific bacteriophage may lead to changes in the composition and biochemical function of a specific bacterial community and may have a profound impact on the global ecosystem.

Bacteriophages are classified based on their genome structure into double-stranded (ds)DNA, single-stranded (ss)DNA, dsRNA, or ssRNA. Due to their comparatively high mutation rates, ssRNA bacteriophages exist as quasispecies, and the evolution speed of ssRNA phages tends to be high [3]. Although it is considered that ssRNA bacteriophages can adapt readily to changes in the environment, it is not clear how quickly they can adapt to a novel environment and/or how many and what types of mutation are required for adaptation. Each environment plays host to various microorganisms that have adapted themselves for optimal growth. Therefore, in general, when bacteriophages adapt to novel environments, they have to not only adapt to the novel environment but also have to adapt to bacterial hosts living there. To elucidate the mechanisms underlying bacteriophage adaptations to the environment in an experimental setting, it is necessary to use a single host strain. This will allow examination of an impact of a single environmental factor on the closed bacteriophage–host system.

Ambient temperature is a critical environmental factor that has had enormous influence on the life history of living organisms on Earth. Thermal adaptation experiments using ssDNA and ssRNA phages have showed parallel evolution in the nucleotide and the detailed relationship between fitness and the underlying genetic changes [4,5,6,7,8,9]. These early studies have focused on thermal adaptation up to 42 °C or 43.5 °C. Other studies focusing on thermal adaptability explored the impact of short-term pulsed exposure of bacteriophages to high temperatures (above 50 °C), followed by culture at a high temperature (43 °C) in nutrient-rich medium, and reported the presence of specific adaptive mutations [10,11]. Because these early studies used ordinary laboratory bacterial strains as a host, they could not grow at temperatures above ~44 °C. Therefore, it is impossible to conduct the thermal adaptation experiment above this temperature. However, true thermal adaptation implies that phages should be capable of growth and replication at higher temperatures, a point that has not yet been fully explored. To investigate the thermal adaptation of phages, it is necessary to use a host strain that is capable of growth at higher temperature as a host. Recently, Kishimoto et al. isolated a strain of thermally adapted *Escherichia coli* that was capable of growing at temperatures up to 46 °C by thermal adaptation evolution experiment [12]. Using this thermally adapted bacterial host, we can explore the potential upper temperature limit for adaptive responses of coliphages.

In our previous study, we determined that Qβ, a ssRNA bacteriophage with a relatively small genome (4217 bases) that replicates within an *E. coli* host, was capable of growth and replication at 43.6 °C; the effects of synonymous and nonsynonymous changes on the fitness and life history of Qβ were evaluated [7,13]. In the present study, we conducted a thermal adaptation experiment with Qβ at temperatures up to 45.3 °C using the aforementioned thermally adapted *E. coli* strain. We showed that experimental evolution of ssRNA phage as a model system is very useful to elucidate its ability to quickly adapt to a novel environment. Accumulation of this fundamental knowledge is critical to predict ssRNA virus evolution.

## 2. Materials and Methods 

### 2.1. Strains and Culture Conditions

The *E. coli* 46L-1 strain was generated from line 1 via adaptation up to 46 °C for 8829 generations [12]. *E. coli* 46L-1 and HB2151 strains [14] were used for constructing the 46L-1F’ strain via conjugation methods reported previously [15], resulting in the introduction of an F plasmid into the thermally adapted 46L-1 strain. The *E. coli* A/λ strain [16] was used as the host strain for the titer assay. The specific growth rate of 46L-1F’ was measured at 37.2 °C, 43.65 °C, (hereafter 43.7 °C), 44.8 °C, 45.3 °C, and 45.9 °C. Straight lines were fitted into semi-logarithmic plots of optical density at 600 nm (OD_600_) to obtain the specific growth rates from each slope. The Qβ 18 mut that was adapted to grow at 43.6 °C as described in our previous reports [7,13] was used as the starting material. The genome of Qβ 18 mut had 17-point mutations and one insertion when compared with the ancestral Qβ sequence. We generated Qβ 18 mut from cDNA using F^–^ *E. coli* DH5α strain using a previously reported method [13]. Briefly, we constructed pACYCQβ_18 mut with 18 specific mutations introduced into the ancestral Qβ cDNA sequence. DH5α/pACYCQβ_18 mut was cultured in Luria–Bertani (LB) broth (10 g/L tryptone, 5 g/L yeast extract, 10 g/L NaCl) with 50 μg/mL kanamycin. After centrifugation, the supernatant was concentrated. The concentrated supernatant with bacteriophage particles was dialyzed against P buffer (50 mM Tris-HCl, pH 7.6, 0.1 M NaCl, 5 mM MgCl_2_, and 0.1 mM EDTA·2Na) and concentrated again. The Qβ 18 mut bacteriophage was stored in 40% glycerol at −20 °C. Modified M63 medium with 1 mM l-leucine and 2.3 mM MgSO_4_·7H_2_O (total 2.5 mM MgSO_4_·7H_2_O) was used for the thermal adaptation experiments as well as the fitness assay. Bacteriophage titration was performed according to standard methods on LB agar and LB soft agar medium [17].

### 2.2. Thermal Adaptation Experiment

Thermal adaptation experiments were performed in three independent lines. Thermal adaptation experiments were first initiated at a temperature of 43.7 °C, which was then raised to 44.1 °C, 44.8 °C, and 45.3 °C in a stepwise manner. Next, to investigate whether the ancestral sequence (Anc(P1)) became dominant in the population, after adaptation at 45.3 °C, the temperature was returned back to 37.2 °C. The culture temperature was increased when the amplification ratio at the holding temperature equilibrated. We used 0.1% bovine serum albumin-coated 15-mL polypropylene centrifuge tubes for preventing adsorption of Qβ to the tube wall. Each serial passage was performed as follows: log phase 46L-1F’ cells grown at each temperature were cultured at a shaking speed of 160 ± 1 rpm and transferred into a fresh medium with dilution to 0.029 or 0.022 OD_600_ (the latter value for 37.2 °C amplification only). The 46L-1F’ cells were cultured for 4 h at the given temperature. After 4 h, the OD_600_ was between 0.126 and 0.210 (approximately 1 × 10^8^ to 2 × 10^8^ CFU/mL calculated from 1 × 10^9^ CFU/mL of OD_600_ = 1). Cells were infected with phages at approximately 9.7 × 10^5^–3.8 × 10^6^ plaque forming units (PFU)/mL from the previous passage. To determine the initial titer, an aliquot was sampled, and free bacteriophages were obtained from the supernatant after centrifugation at 13,400× *g* for 1 min at room temperature. The bacteriophage-infected cultures were grown for approximately 5 h and divided into three aliquots; one aliquot was used for titration of free bacteriophage particles after centrifugation as noted above; the second was used to determine OD_600_, whereas the third was used for preparing the −80 °C frozen (15% glycerol) stock. Free phage was titrated and stored at 4 °C for performing infection in the subsequent serial passage. The replication generations (*g*) of Qβ were calculated as the cumulative generations of each passage, (*N*_5_/*N*_0_) = 2*^g^*, where *N*_5_ and *N*_0_ represent the free-phage concentration (PFU/mL) at 5 h and 0 h, respectively, and *g* represents the replication generation.

### 2.3. Fitness Analysis

The fitness of Qβ 18 mut and 12 endpoint populations after thermal adaptation was evaluated at 37.2 °C, 43.7 °C, 44.8 °C, and 45.3 °C using 46L-1F′ as the host strain. The endpoint populations were designated as 43.7_1, 43.7_2, 43.7_3, 44.8_1, 44.8_2, 44.8_3, 45.3_1, 45.3_2, 45.3_3, 37.2_r1, 37.2_r2, and 37.2_r3 with the numbers before and after the underscore representing the passage temperature and passage line, respectively. The letter “r” was added to distinguish these variants with the 37.2 °C adaptation in our previous study. The 46L-1F′ strain was cultured at 37.2 °C, 43.7 °C, 44.8 °C, and 45.3 °C as described above, and the bacterial cells were infected with approximately 4.1 × 10^4^–2.8 × 10^6^ PFU/mL of Qβ. Free bacteriophage concentration was determined as described above immediately after inoculation (0 h) and approximately 5 h after infection. Relative fitness was calculated as *x* = log_10_ (*N*_5_/*N*_0_), where *N*_5_ and *N*_0_ represent free bacteriophage concentrations (PFU/mL) at 5 h and 0 h, respectively. Qβ 18 mut was used as a control for each experiment.

### 2.4. Genome Sequencing of Qβ

The RNA genomes of the Qβ populations were derived from approximately 1 × 10^9^–1 × 10^10^ PFU of the variants of the aforementioned 12 populations. RNA genome was extracted using the QIAamp Viral RNA mini kit (Qiagen, Hilden, Germany) according to the manufacturer’s instructions. To analyze the full-length RNA genome sequence, samples were prepared using a previously described method [15]. Briefly, a poly(A) sequence was added to the 3′ end of each RNA genome, facilitating cDNA synthesis using a poly(T) primer; poly(A) was added to the 3′ end of the cDNA. PCR was performed using Phusion High-Fidelity DNA polymerase (New England Biolabs, Ipswich, MA, USA) or Pfu Ultra II Fusion HS DNA polymerase (Agilent Technologies, Santa Clara, CA, USA); the Qβ genome was divided into six regions using the primers shown in Table S1. When a double peak appeared in the sequencing chart, we measured the height of the peak and calculated the ratio of mutated to ancestral sequence. The secondary structure of each RNA genome was estimated using RNAfold [18].

### 2.5. Statistical Analysis

Relative fitness were compared using one-way analysis of variance (ANOVA), and a *p*-value of 0.01 was taken to indicate statistical significance [19].

## 3. Results

### 3.1. Experimental Evolution

**Figure 1:**
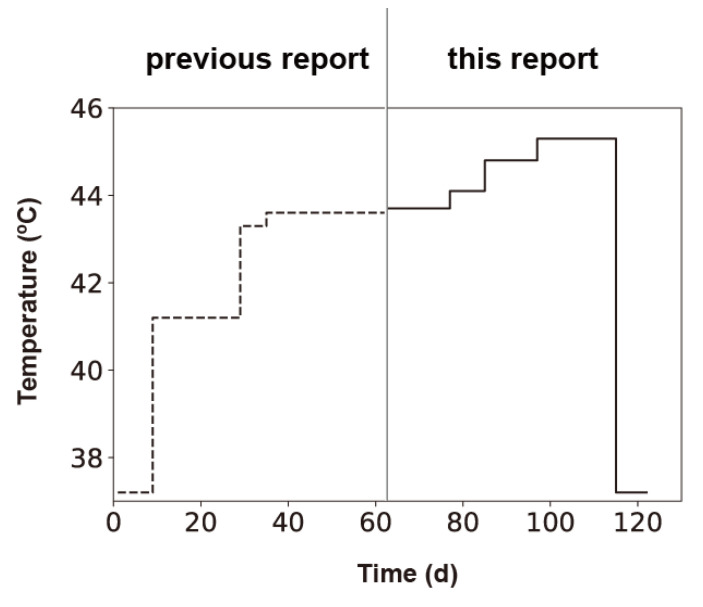
Thermal profile of the adaptation experiments. Thermal adaptation was initiated at 37.2 °C; culture temperatures were increased in a stepwise manner to 45.3 °C. After adaptation to 45.3 °C, populations were then returned to 37.2 °C to investigate whether Qβ can revert to its ancestral sequence. The adaptation process for temperatures ranging from 37.2 to 43.6 °C has been described previously [7].

We conducted thermal adaptation experiments between 43.7 and 45.3 °C with three independent lines (Figure 1). In our previous report, we conducted thermal adaptation experiments between 37.2 and 43.6 °C with three independent lines using *E. coli* 43BF’ as the host strain [7,13]. Qβ 18 mut was one of the three replicates that had adapted to 43.6 °C; the genome of this variant had 17-point mutations and one insertion when compared with the ancestral Qβ sequence. In the present study, we prepared Qβ 18 mut from cDNA and used it as the starting phage. To monitor Qβ thermal adaptation, we used the thermally adapted *E. coli* strain 46L-1F’. Because the growth of Qβ depends on the growth of the *E. coli* host, we analyzed the specific growth rate of *E. coli* 46L-1F’ strain at temperatures of 37.2 °C, 43.7 °C, 44.8 °C, 45.3 °C, and 45.9 °C. The specific growth rates of *E. coli* 46L-1F’ were 0.42 ± 0.007, 0.42 ± 0.008, 0.42 ± 0.015, 0.39 ± 0.014, and 0.31 ± 0.011 h^−1^ at 37.2 °C, 43.7 °C, 44.8 °C, 45.3 °C, and 45.9 °C, respectively. The specific growth rate of *E. coli* 46L-1F’ was almost identical between 37.2 and 44.8 °C but decreased by 7% and 26% at 45.3 °C and 45.9 °C, respectively. Therefore, we conducted our thermal adaptation experiments at temperatures up to 45.3 °C.

**Figure 2:**
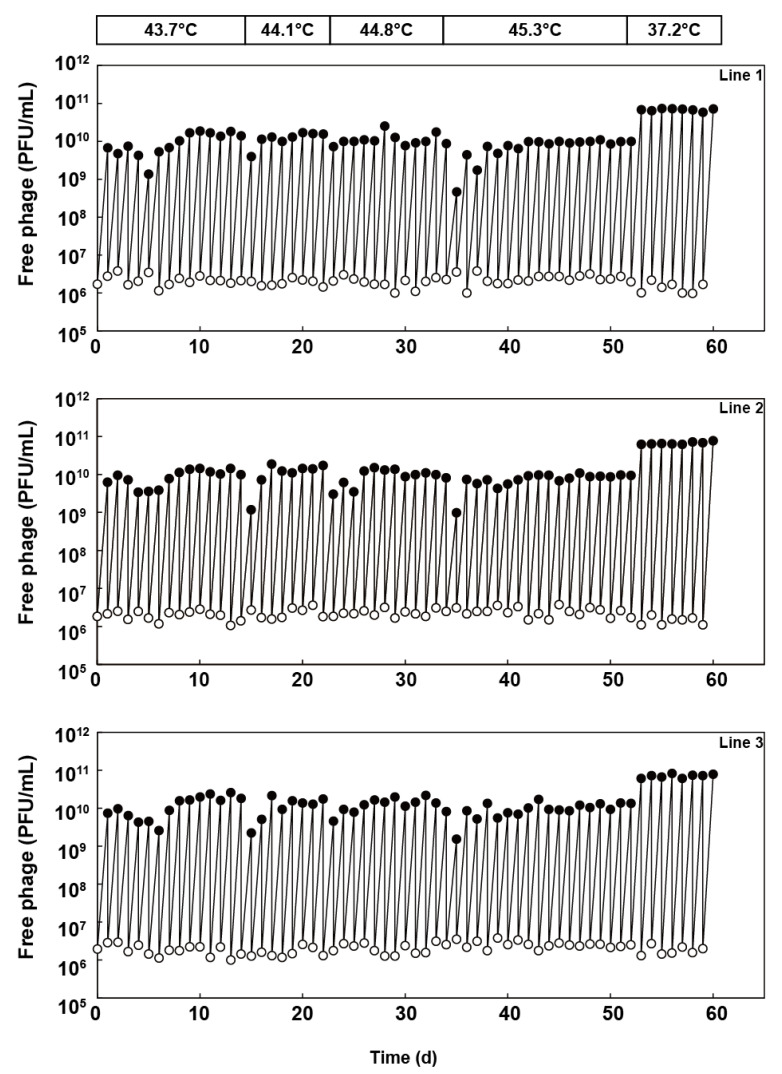
Population dynamics of the three independent Qβ lines in thermal adaptation. Population dynamics of the free-phage densities (PFU/mL) of lines 1, 2, and 3 are as shown. The free-phage densities immediately after (0 h) and at 5 h after infection were measured each day using the method described in the Materials and Methods section.

Qβ 18 mut bacteriophage was divided into three separate lines and passaged in the *E. coli* 46L-1F’ strain, in which the culture temperature was increased sequentially as follows: 43.7 °C, 44.1 °C, 44.8 °C, and 45.3 °C. Free phage was isolated from the previous day’s culture, diluted, and used to infect a fresh *E. coli* 46L-1F’ strain at the logarithmic growth phase. The diluted phage was added to an initial density of 1 × 10^6^ to 4 × 10^6^ PFU/mL, which was equivalent to an effective population size (*N_e_*) of approximately 2.5 × 10^6^ to 1 × 10^7^ PFU, which was determined by multiplying the free-phage density immediately after inoculation of 2.5 mL of culture. Initially, we passaged Qβ at 43.7 °C for 14 days (i.e., equivalent to 167, 169, and 175 generations for lines 1, 2, and 3, respectively). After 14 days, we performed thermal adaptation at 44.1 °C, 44.8 °C, and 45.3 °C for 8 (equivalent to 100, 97, and 101 generations for line 1, 2, and 3), 12 (equivalent to 151, 143, and 151 generations for line 1, 2, and 3), and 18 days (equivalent to 206, 207, and 211 generations for lines 1, 2, and 3), respectively (Figure 2). Population dynamics revealed that at every temperature shift, there was a rapid recovery in the amplification ratio in the primary 1–5 days and equilibrated after an initial decrease in yield. Qβ was capable of replication at 45.3 °C within 114 days (including 52 days in this study and 62 days from 37.2 to 43.6 °C in a previous study [7]) (Figure 1). 

Next, to investigate whether the ancestral sequence (Anc(P1)) became dominant in the population, we changed the culture temperature from 45.3 to 37.2 °C, which is the temperature that permits optimum growth of the original Qβ Anc(P1). We passaged the 45.3 °C-adapted Qβ population at 37.2 °C for 8 days (equivalent to 124, 124, and 122 generations for lines 1, 2, and 3, respectively).

The endpoint Qβ populations at 43.7 °C, 44.8 °C, 45.3 °C, and 37.2 °C were used for further genotypic and phenotypic analyses. 

### 3.2. Fitness of Thermally Adapted Populations

**Figure 3:**
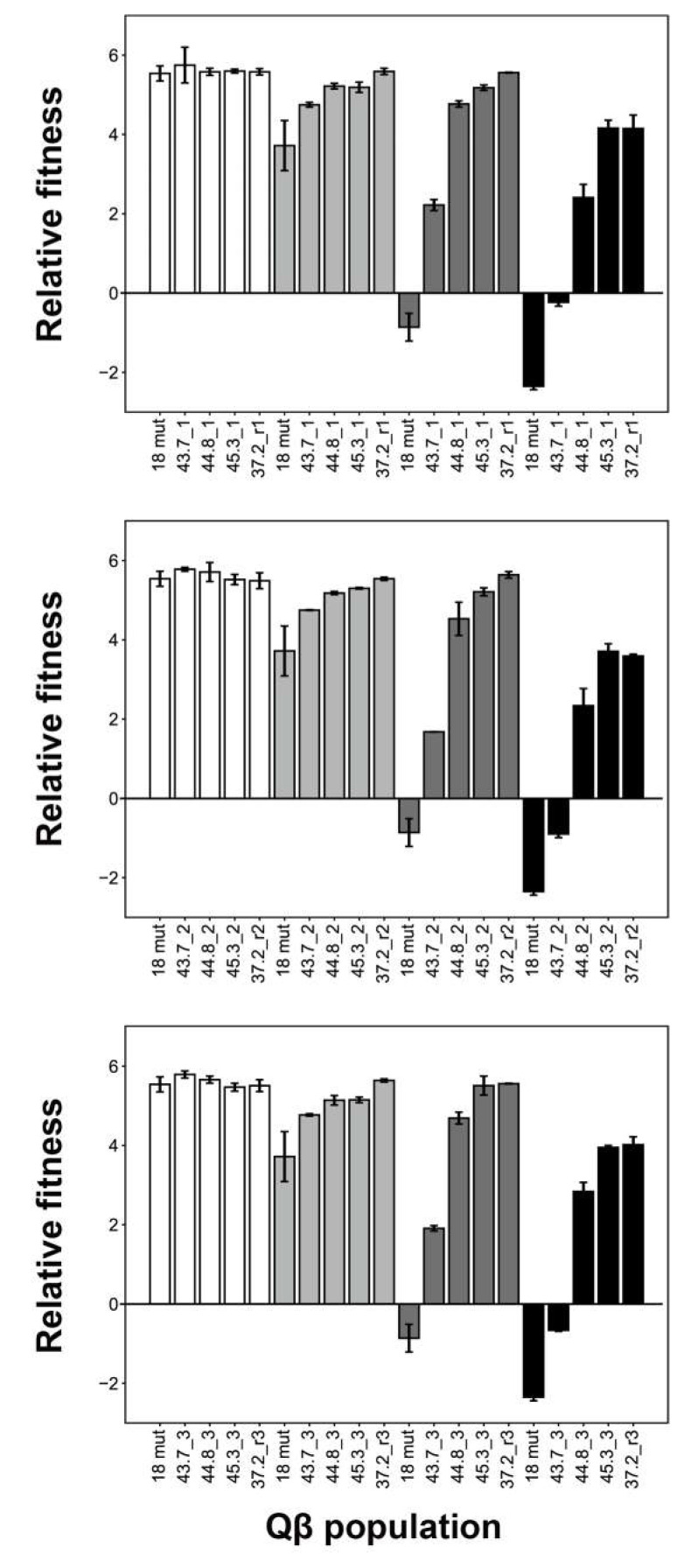
Relative fitness of Qβ 18 mut and populations that evolved under conditions of elevated temperature. Relative fitness was measured at 37.2 °C, 43.7 °C, 44.8 °C, and 45.3 °C for the Qβ 18 mut and endpoint 43.7 °C-, 44.8 °C-, 45.3 °C-, and 37.2 °C-adapted populations. Upper, middle, and lower frames demonstrate the relative fitness of lines 1, 2, and 3, respectively. Data are presented as means ± standard deviations (*n* = 2–14).

We performed fitness analysis at 37.2 °C, 43.7 °C, 44.8 °C, and 45.3 °C using the 12 endpoint populations noted above (Figure 3). Relative fitness was calculated as the ratio of free bacteriophage concentrations (PFU/mL) at 5 h and 0 h, respectively. Fitness analysis revealed that at 45.3 °C, the thermally adapted Qβ populations could grow at 45.3 °C but 18 mut could not grow. At 37.2 °C, there were no significant differences among any of the evolved populations and Qβ 18 mut (one-way ANOVA, *F*_4,16_ = 0.41, *p* = 0.80: line 1; *F*_4,16_ = 1.27, *p* = 0.32: line 2; *F*_4,16_ = 1.81, *p* = 0.18: line 3). These results showed that the 45.3 °C-adapted Qβ population did not demonstrate decreased fitness at 37.2 °C. At 43.7 °C, the 44.8 °C- and 45.3 °C-adapted populations presented a tendency toward improved fitness compared with that of the 43.7 °C-adapted populations. These results indicate that thermal adapted Qβ broadened the temperature range for growth.

After adaptation at 45.3 °C, the culture temperature was reverted back to 37.2 °C. Interestingly, the fitness of 37.2_r1, _r2, and _r3 at 45.3 °C did not decrease compared with that of the 45.3 °C-adapted populations. In addition, 37.2_r1, _r2, and _r3 tended to exhibit improved fitness compared with that of the 43.7 °C-, 44.8 °C-, and 45.3 °C-adapted populations grown at 43.7 °C and 44.8 °C.

### 3.3. Molecular Evolution of Thermally Adapted Qβ

We determined the whole genome sequences of endpoint populations at 43.7 °C, 44.8 °C, 45.3 °C, and 37.2 °C (Table 1 and Table 2). As noted earlier, Qβ 18 mut had 1-bp insertion in a non-coding region at the 5′ end of the genome [7,13]; this resulted in an increase in the genome size to 4218 bases. We determined the sequence as the representative sequence of the population by extracting RNA genome from the population. We compared the sequences of the endpoint populations with Qβ 18 mut or Qβ Anc(P1). Among those adapted to 43.7 °C, three nonsynonymous mutations were observed at the same positions in the genome of all three lines; these included A1781C, U3784C, and C3879G/A. Other mutations (1–4) were observed in each of the three lines. Among the Qβ populations adapted to 44.8 °C, additional 7-, 13-, and 8-point mutations were added to lines 1, 2, and 3, respectively; 3, 0, and 2 mutations became undetectable. At 45.3 °C adaptation, additional 13-, 6-, and 10-point mutations were added to lines 1, 2, and 3, respectively, and 4, 7, and 0 mutations became undetectable. In total, we identified substitutions at 20, 16, and 21 sites in lines 1, 2, and 3, respectively, which were associated with the stepwise adaptation process from 43.7 to 45.3 °C. Overall, these represented 38, 34, and 39 substitutions from the ancestral Qβ Anc(P1) in lines 1, 2, and 3, respectively. These results suggest that Qβ could adapt to these elevated temperatures with only point mutations; these mutations account for 0.8%–0.9% of the total RNA genome (Table 1 and Table 2). In the 45.3 °C-adapted population, four mutations, i.e., A52G, G1494A, C3659U, and C3879G, were observed in all three lines. However, these substitutions occurred at 43.7 °C or 44.8 °C. During the 45.3 °C adaptation, nineteen additional mutation sites first appeared, however, these sites were not common to all three lines. In addition, mutations introduced during this adaptation have a tendency to increase the frequency in NCR and A1 but not randomly in all the genes (Table 3).

We also analyzed the sequences of 37.2_r1, 37.2_r2, and 37.2_r3. In total, 3-, 4-, and 8-point mutations were introduced, whereas the frequency of 1, 1, and 5 mutations decreased in each population in line 1, 2, and 3, respectively, during the reverse 37.2 °C adaptation (Table 1 and Table 2).

## 4. Discussion

In this study, we explored the adaptation process of ssRNA bacteriophage Qβ via stepwise increases to the highest known growth temperature, 45.3 °C. We showed that Qβ can grow and replicate at this temperature within 52 days (616 generations) when the Qβ 18 mut variant is used as the starting material and within 114 days (1238 generations) when ancestral Qβ, which has an optimum growth temperature of ~37 °C, was used as the starting material. The 45.3 °C-adapted population had at most 21 substitutions from Qβ 18 mut and 39 substitutions from ancestral Qβ. Overall, temperature adaptation results in mutations in 0.8–0.9% of the Qβ genome. 

Although the growth of *E. coli* depends on the culture medium, ordinary laboratory strains cannot grow in minimal media at temperatures greater than 43 °C [20]. Furthermore, the rate of polypeptide synthesis begins to drastically decrease at temperatures greater than 44 °C [21]. However, the experiments featured in the present study used the *E. coli* 46L-1F’ strain that can efficiently grow at temperatures ranging between 37.2 and 45.3 °C. This technical advance made it possible to evaluate Qβ adaptation to greater thermal changes. We found that Qβ adapted to growth and replication at 45.3 °C had an overall increased temperature range because these populations could grow with equivalent fitness at 37.2 °C. Intriguingly, even though we returned the 45.3 °C-adapted populations to 37.2 °C for 122–124 generations, little to no decrease in fitness was observed. These results clearly indicate that Qβ gained the potential for growth at higher temperatures without showing trade-off in the lower ranges.

Previously, Qβ was passaged at 37.2 °C for ~120 generations in three replicates from Anc(P1) using the same method [7]. Kashiwagi et al. (2014) observed C2249U in all the three replicates and another 1-point mutation in the 5′ noncoding region (G4G/A) in one of three replicates [7]. Therefore, in this study, most of the mutations we observed might be introduced upon thermal adaptation. However, as reported by Singhal et al., (2017) with regard to the importance of considering the effects of multiple selective pressures, even in environments where a single factor is changed [10], further analysis is required to determine the effects of each mutation on the fitness increase at 45.3 °C. In addition, we observed the introduction of some mutations at the 37.2 °C passage with the 45.3 °C-adapted populations. To compare the result with the previous 37.2 °C passage starting from Anc(P1), the mutation fixation number per generation was counted using polymorphic sites and monomorphic sites and was found to be 0.5 and 1, respectively. The mutation number per generation of the 37.2 °C passage using 45.3 °C-adapted populations (3.4 × 10^–2^ mutations/generation) was larger than that of the 37.2 °C passage starting from Anc(P1) (5.7 × 10^–3^ mutations/generation). This difference might be because of the difference in the sequences used as the starting phage population. 

The Qβ genome encodes four proteins: A2, coat, A1, and β subunit for Qβ replicase. A2 is a multifunctional protein that binds the F pili of *E. coli* and lysis function via binding with MurA [22,23,24,25,26]. Cryo-electron microscopic analysis has clearly revealed that the β-sheet-rich region of A2 protrudes from the capsid, while the α-helix-rich region is within the capsid [27]. Here we found two amino acid substitutions, Ser401Arg and Phe411Cys, in two of three lines as well as two amino acid substitutions, Val132Ala and His385Arg, in one of three lines. Mapping these mutational positions on the A2 structure (Protein Data Bank 5MNT) reveals the following: (i) Ser401 is located near the position where coat and A2 interact [28] (Figure S1a). (ii) Phe411 is located in α9 and is close to the RNA-binding region [29] (Figure S1b). (iii) Val132 and His385 are closely located at the protruding region (Figure S1a). The region between the 30th and 120th amino acids of the N-terminus of A2 is in contact with MurA for cell lysis [28], and no mutations, except Glu30Asp, are observed in this region. In coat protein, we observed Val50Ile substitution in all three lines. Asn30, Thr49, Ser51, and Gln65 produce an adenine-binding pocket to fit the A nucleotide of the operator of the β subunit gene [30]. When it is mapped on the structure (Protein Data Bank 4L8H), Val50 is also located near the operator sequence (Figure S1c), indicating that this substitution might be related to the translation of the β subunit. When the coat stop codon is suppressed at low probability, A1 is produced as the read-through protein. Thus, A1 has an additional 197 amino acids in its C-terminus [31]. We observed Lys145Asn in lines 2 and 3 and Ser144Leu in line 3; however, the Ser144 and Lys145 positions in A1 were missing in the crystal structure in Protein Data Bank 3RLC. The functional effects of these amino acid changes are unclear. Next, Qβ replicase comprises a β subunit from Qβ, translational elongation factors EF-Tu and EF-Ts, and ribosomal protein S1 from *E. coli* [32,33]. The β subunit structure comprises finger, thumb, and palm domains [34]. We observed eight nonsynonymous mutations in the β subunit gene. Ala33Val, Met75Leu, and Ile477Thr were found in two of three lines, while Leu509Val (mutation 3879) was found in all three lines. Tyr510 interacts with the 5’ terminus of newly synthesized RNA in the processive elongation stage, and Tyr510 mutation decreases replication activity [35]. Since Leu509Val is the next amino acid of Tyr510, this amino acid substitution might be related to replication activity. When we mapped on the structure (Protein Data Bank 3AVT), Val141Ala, Asp347Asn, and Ser350Pro were located near the region to interact with S1 (Figure S1d) [36] and Ala33, Met75, and Ile477 were located on the thumb domains. The functional effects of these amino acid changes are unclear.

The frequency of both nonsynonymous and synonymous mutations increased in the population (Table 1). The Qβ RNA genome is a multifunctional RNA that acts as an mRNA-encoding protein, a genome replication template, and regulatory RNA for controlling the level and timing of protein expression in the Qβ life cycle [22]. In addition, the secondary structure of the ssRNA genome has important functions with respect to bacteriophage formation. The RNA genome has to be compact to be packaged appropriately into the capsid. Furthermore, it should have specific contacts with the coat protein inside the capsid, and should be able to recruit coat proteins to assemble the capsid [27,37,38]. The propagation processes of synonymous mutations have been extensively investigated by experimental evolution of ssDNA and ssRNA viruses, in which synonymous mutations and mutations in intergenic regions were fixed during adaptation to elevated temperatures [4,5,6]. In Qβ thermal adaptation, not only nonsynonymous mutations but also synonymous mutations contributed to fitness increase [7,13]. Therefore, some, and not all, synonymous mutations introduced in this study may have been adaptive. The secondary structures predicted for the thermally adapted RNA sequences are shown in Figure S2. We introduced mutations observed at over 90% frequency in each population. We observed the slight changes in the secondary structure of 44.8_1, 45.3_1, and 37.2_r1 or 45.3_2 and 37.2_r2, which correspond to the beginning region of the β subunit gene or the region between the A1 gene and the beginning of the β subunit gene. Some nonsynonymous mutations became frequent and were lost later, possibly as a result of the following mechanisms: clonal interference, epistasis, or hitchhiking when introducing additional adaptive mutations into the population [5].

To understand the mechanisms underlying adaptation and speciation, the concept of fitness landscape has been proposed [39,40]. Fitness landscape is essentially the relationship between genotype and response or fitness for a given environment. The direction of evolution may be predicted with a strong understanding of the shape of a given fitness landscape [41]. Theoretical models, such as the NK model, Mt. Fuji model, and rough Mt. Fuji model, have been presented in support of this concept [42,43,44]. Several studies that have focused on the evolution of proteins, RNA aptamers, and ribozymes have been presented with respect to the shape of fitness landscape [45,46,47,48,49]. For example, in a comprehensive study of short-length RNA, fitness peaks were isolated from one another [50]. In the present study, we found that when populations adapted to 45.3 °C were readapted at 37.2 °C, the fitness at the higher temperature was maintained. When we assumed that the mutations present in over 90% of the sequences in a given population were fixed or nearly fixed, we found that most of the mutational sites were polymorphic (Table 1). These results suggest that the fitness landscape of Qβ at 45.3 °C is flat rather than rugged. 

In experiments performed with thermally adapted *E. coli*, the Qβ bacteriophage adapted to the higher temperature (45.3 °C) within 114 days (~1260 generations); interestingly, *E. coli* required almost 537 days (equivalent to 7780 generations) to reach the same endpoint [12]. These results underscore our observations regarding the rapid adaptation of RNA phages to new environments. Bacteriophages are critical components of the global ecosystem. Therefore, additional studies focusing on the understanding of their capacity for adaptation are warranted. Future studies might include DNA as well as RNA bacteriophages. Studies like these are not only critical for the field related to evolutionary biology but also for the field of control of epidemic diseases by viruses for humans, livestock, and plants.

## Figures and Tables

**Table 1:** Nucleotide sequences of the Anc(P1), 18 mut and evolved phage genomes.

Sequence Identity in the Indicated Phage Populations ^a^														Gene and/or Site	Genome Position	Nucleotide in:		Gene Position ^b^	Codon Change	Amino Acid Change
Anc(P1)	18 mut	43.7_1	44.8_1	45.3_1	37.2_r1	43.7_2	44.8_2	45.3_2	37.2_r2	43.7_3	44.8_3	45.3_3	37.2_r3			Anc(P1)	Evolved Population			
	+	+	+	+	+	+	+	+	+	+	+	+	+	UTR	4	G	A			
	+	+	+	+	+	+	+	+	+	+	+	+	+	UTR	14		A+1 insertion			
											+	+	+	UTR	39	U	C			
			68				25							UTR	47	G	A			
					45									UTR	51	A	G			
				72	22		23	+	87		+	+	+	UTR	52	A	G			
							28							UTR	54	A	G			
				30			26	+	+					A2	141	C	U	81(26)	GAC→GAU	
	+	+	+	+	+	+	+	+	+	+	+	+	+	A2	153	A	C	93(30)	GAA→GAC	Glu→Asp
	+	+	+	+	+	+	+	+	+	+	+	+	+	A2	192	U	C	132(43)	CGU→CGC	
												20		A2	228	U	C	168(55)	AAU→AAC	
								57	34					A2	420	C	U	360(119)	ACC→ACU	
								57	34					A2	458	U	C	398(132)	GUU→GCU	Val→Ala
								+	87					A2	831	U	C	771(256)	GUU→GUC	
							24	+	+					A2	834	U	C	774(257)	GCU→GCC	
											+	+	+	A2	852	G	A	792(263)	GGG→GGA	
	+	+	+	+	+	+	+	+	+	+	+	+	+	A2	905	A	G	845(281)	GAA→GGA	Glu→Gly
											+	+	+	A2	1065	A	G	1005(334)	CAA→CAG	
	+	+	+	+	+	+	+	+	+	+	+	+	+	A2	1088	A	G	1028(342)	GAU→GGU	Asp→Gly
													35	A2	1122	G	A	1062(353)	CGG→CGA	
				88	32									A2	1158	G	A	1098(365)	GGG→GGA	
				+	+									A2	1217	A	G	1157(385)	CAU→CGU	His→Arg
			76										26	A2/S-site	1251	U	G / (37.2_r3 C)	1191(396)	CUU→CUG/CUC	
	+	+	+	+	+	+	+	+	+	+	+	+	+	A2/S-site	1257	C	U	1197(398)	ACC→ACU	
							26	40				26		A2/S-site	1266	U	G	1206(401)	AGU→AGG	Ser→Arg
	+	+	+	+	+	+	+	+	+	+	+	+	+	A2/S-site	1281	U	C	1221(406)	GUU→GUC	
				+	+							59	75	A2/S-site	1295	U	G	1235(411)	UUU→UGU	Phe→Cys
	+	+	+	+	+	+	+	+	+	+	+	+	+	A2/S-site	1312	G	A	1252(417)	GUA→AUA	Val→Ile
	+	+	+	+	+	+	+	+	+	+	+	+	+	Coat /A1	1371	G	A	28(9)	GGU→AGU	Gly→Ser
	+	+	+	+	+	+	+	+	+	+	+	+	+	Coat /A1	1400	U	C	57(18)	ACU→ACC	
			22	+	+		37	+	+			24	21	Coat /A1	1494	G	A	151(50)	GUU→AUU	Val→Ile
							23	+	+			22	28	Coat /A1	1604	C	U	261(86)	CGC→CGU	
		47	+	+	+		62							A1	1775	G	U	432(143)	GGG→GGU	
												21		A1	1777	C	U	434(144)	UCA→UUA	Ser→Leu
		46				49	36	+	+	75	+	+	+	A1	1781	A	C	438(145)	AAA→AAC	Lys→Asn
		20												A1	1831	G	A	488(162)	GGU→GAU	Gly→Asp
													24	A1	1872	G	A	529(176)	GUU→AUU	Val→Ile
			22											A1	1893	A	G	550(183)	AAC→GAC	Asn→Asp
													31	A1	1956	A	C	613(204)	AAA→CAA	Lys→Gln
								21	36				43	A1	2006	U	G	663(220)	AGU→AGG	Ser→Arg
									23				40	A1	2016	U	G/ (37.2_r3 C)	673(224)	UUC→GUC /CUC	Phe→Val/Leu
						32	76							A1	2061	U	C	718(239)	UAU→CAU	Tyr→His
											+	+	+	A1	2078	G	A	735(244)	CAG→CAA	
											22	45	24	A1	2087	U	C	744(247)	CGU→CGC	
				+	+									A1	2111	G	A	768(255)	GAG→GAA	
	+	+	+	+	+	+	+	+	+	+	+	+	+	A1	2201	C	U	858(285)	GCC→GCU	
				+	+									A1	2246	C	U	903(300)	UCC→UCU	
±	+	+	+	+	+	+	+	+	+	+	+	+	+	A1	2249	C/U	U	906(301)	AGC→AGU	
				89	38									A1	2291	U	C	948(315)	ACU→ACC	
		23						79	46		+	+	+	β -subunit	2452	C	U	101(33)	GCC→GUC	Ala→Val
												22		β -subunit	2462	A	G	111(36)	UUA→UUG	
		39	+	+	+									β -subunit	2534	G	A	183(60)	GGG→GGA	
				+	22					52	+	+	+	β -subunit/M-site	2577	A	C/ (45.3_1, 37.2_r1 U)	226(75)	AUG→CUG/UUG	Met→Leu
				+	+									β -subunit/M-site	2623	U	C	272(90)	GUU→GCU	Val→Ala
	+	+	+	+	+	+	+	+	+	+	+	+	+	β -subunit/M-site	2748	A	C	397(132)	AGA→CGA	
				+	+									β -subunit/M-site	2753	A	G	402(133)	AAA→AAG	
	+	+	+	+	+	+	+	+	+	+	+	+	+	β -subunit/M-site	2776	U	C	425(141)	GUU→GCU	Val→Ala
								28	30			22	26	β -subunit	2879	G	A	528(175)	CCG→CCA	
													21	β -subunit	2949	A	G	598(199)	AUU→GUU	Ile→Val
				84	26									β -subunit	3032	U	C	681(226)	GGU→GGC	
							37							β -subunit	3047	C	U	696(231)	UUC→UUU	
									31					β -subunit	3086	U	G	735(244)	CGU→CGG	
							33							β -subunit	3206	C	U	855(284)	GCC→GCU	
				+	+									β -subunit	3260	A	G	909(302)	AGA→AGG	
												37		β -subunit	3393	G	A	1042(347)	GAC→AAC	Asp→Asn
	+	+	+	+	+	+	+	+	+	+	+	+	+	β -subunit	3402	U	C	1051(350)	UCG→CCG	Ser→Pro
					35									β -subunit	3455	C	A	1104(367)	GUC→GUA	
			21	+	+		24		28			24	32	β -subunit	3545	C	U	1194(397)	GGC→GGU	
					30									β -subunit	3653	A	G	1302(433)	ACA→ACG	
			45	+	+		+	+	+		+	+	+	β -subunit	3659	C	U	1308(435)	GAC→GAU	
		54	+	+	+	37	84	+	+	21				β -subunit	3784	U	C	1433(477)	AUC→ACC	Ile→Thr
			24											β -subunit	3809	G	A	1458(485)	GGG→GGA	
		47	+	+	+	38	27	+	+	27	+	+	+	β -subunit	3879	C	G / (43.7_2 A, 44.8_2 A and G)	1528(509)	CUC→GUC/AUC	Leu→Val /Ile
									28	40				β -subunit	3903	C	U	1552(517)	CUC→UUC	Leu→Phe
	+	+	+	+	+	+	+	+	+	+	+	+	+	β -subunit	3931	U	C	1580(526)	CUU→CCU	Leu→Pro
	+	+	+	+	+	+	+	+	+	+	+	+	+	β -subunit	4004	G	A	1653(550)	ACG→ACA	
													20	UTR	4193	A	U			

^a^ Sequence identities are indicated as follows: blank represents the same sequence as the ancestral sequence (Anc(P1)), + represents changes in >90% of the sequences, and values represent the percentage of sequences with changes from their ancestral sequence. Light green-painted lines represent the mutational position carried by 18 mut and all the thermal-adapted populations. Pink-painted lines represent the mutational position carried by all the three 45.3 °C-adapted populations. Orange-painted lines represent the mutational position carried two of the three replicates of the 45.3 °C-adapted population. ^b^ Gene position was counted from A of the start codon (AUG) of each gene as position 1. The numbers in parentheses represent amino acid positions of each protein in which the position of second codon was counted as position 1.

**Table 2:** Number of mutations introduced with adaptation to increased temperature and numbers that reverted back to the original sequence.

	Classification *	Thermal Adaptation Temperature											
		43.7 °C			44.8 °C			45.3 °C			37.2 °C		
		L1	L2	L3	L1	L2	L3	L1	L2	L3	L1	L2	L3
Mutations introduced	18 mut→Hetero	7	4	5	7	12	1	5	5	10	3	4	8
	Hetero→Major	0	0	0	4	0	3	3	8	0	0	0	0
	18 mut→Major	0	0	0	0	1	7	8	1	0	0	0	0
Mutations reverted	Hetero→Anc(P1)	0	0	0	3	0	2	4	7	0	1	1	5
	Major→Hetero	0	0	0	0	0	0	0	0	0	1	2	0
	Major→Anc(P1)	0	0	0	0	0	0	0	0	0	0	0	0

L1, L2, and L3 represent line 1, 2, and 3, respectively. * Hetero or Major were determined as the ratio of mutant/ancestral sequences in the adapted population as ratio 10% < ratio < 90% or ratio ≥ 90%, respectively.

**Table 3:** Tendency of mutations within individual Qβ genes.

Gene	Gene Length (Bases)	No. of Mutational Positions *	Mutations/Gene Length
NCR	192	4 (6)	0.021 (0.031)
A2	1263	13 (20)	0.010 (0.016)
Coat	402	2 (4)	0.005 (0.010)
A1	591	12 (14)	0.020 (0.024)
β	1770	18 (23)	0.010 (0.013)

* The value outside the parentheses is the number of mutational positions from 18 mut to 45.3 °C adaptation in this study; the value within the parentheses is the number of mutational positions of 45.3 °C adaptation with 18 mut. The number of mutational positions was calculated as the difference from 18 mut sequence.

## Supplementary Materials

The following are available online at https://www.mdpi.com/1999-4915/12/6/638/s1, Figure S1: Positions of amino acid mutations in the structure, Figure S2: Secondary structure of starting Qβ and thermal adapted Qβ genomes, and Table S1: List of templates, primers, and polymerase used for Qβ genome sequencing.
