# Supplementary material for: The Single-Stranded RNA Bacteriophage Qβ Adapts Rapidly to High Temperatures: An Evolution Experiment
Source: Viruses. 2020 Jun 12;12(6):638. doi: 10.3390/v12060638 (PMC7354602; doi:10.3390/v12060638)
Supplement: Supplementary file 1 [file viruses-12-00638-s001.pdf]

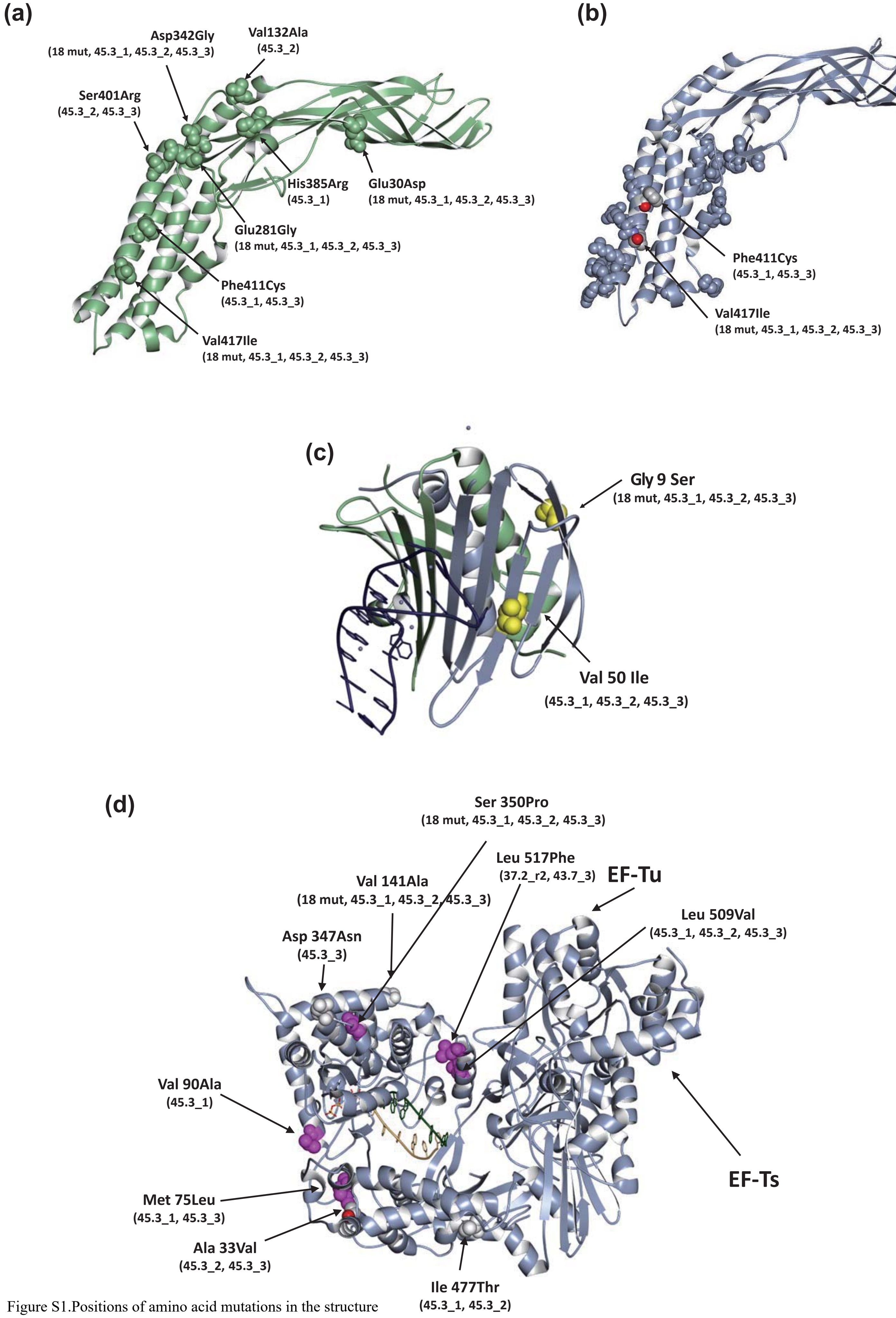


Figure S1.Positions of amino acid mutations in the structure

The amino acid position and the population name in which the mutation was observed is in parentheses. (a) The mutational positions in A2 were mapped on protein data bank 5MNT. (b) The mutational positions in A2 were mapped in the same manner as (a), but the positions shown in gray filled circles represent the amino acid that interacts with the RNA genome [29]. (c) The mutational positions in coat protein were mapped on protein data bank 4L8H. (d) The mutational positions in β-subunit of Qβ replicase with the structure of EF-Tu and EF-Ts (protein data bank 3AVT).

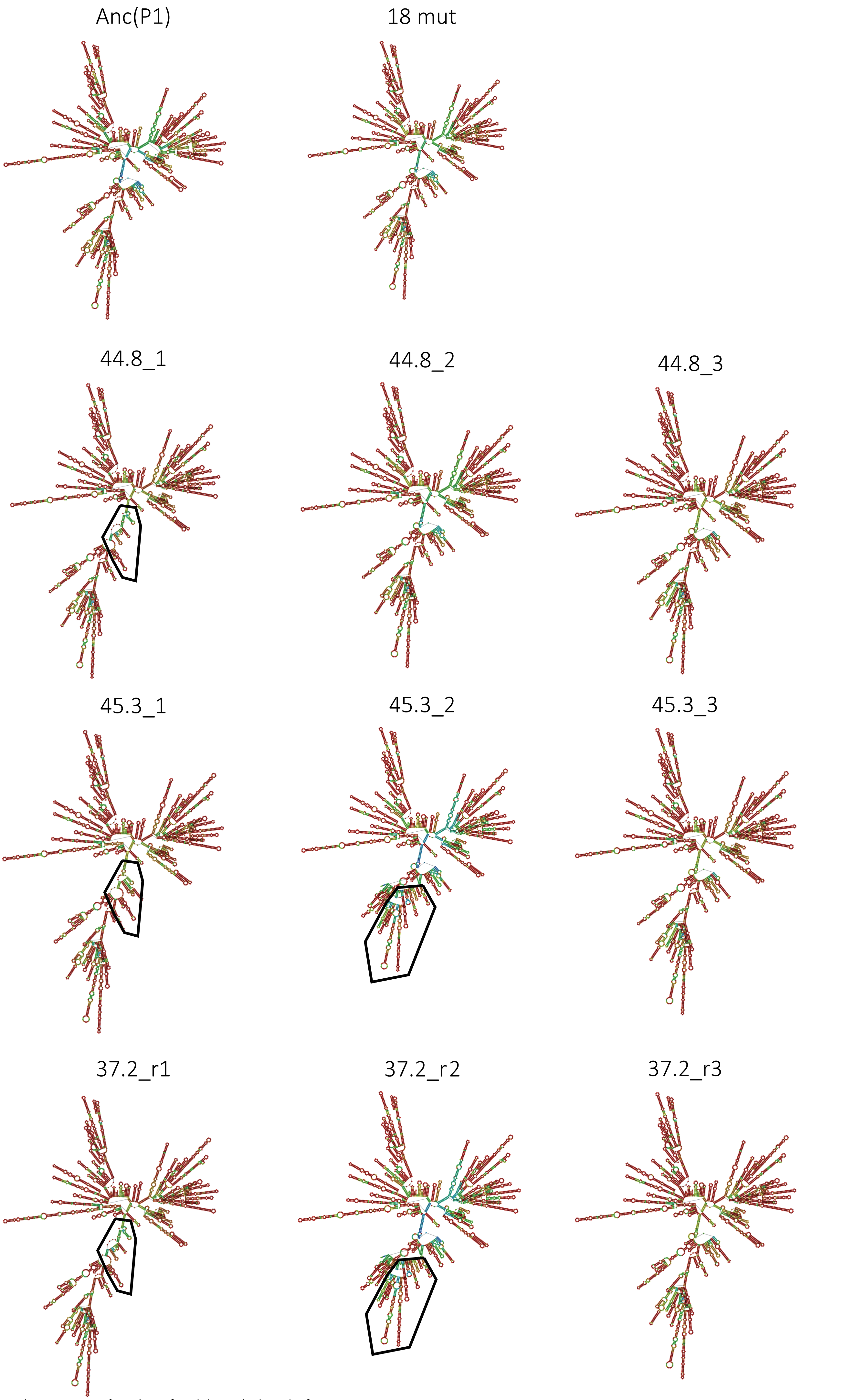


Figure S2. Secondary structure of starting Qβ and thermal adapted Qβ genomes
Anc(P1) is a starting phage cultured from 37.2°C in the previous study [7]; 18 mut is a starting phage in this study. Minor differences were observed in the closed region.

**Table S1. List of templates, primers, and polymerase used for Qβ genome sequencing.**

| PCR products | PCR product preparation | | | | Sequencing | |
|---|---|---|---|---|---|---|
| | Template | Primer name | Primer sequence (5'→3') | DNA polymerase | Primer name | Primer sequence (5'→3') |
| Qt & Qi & seq_3368 product | 2nd strand cDNA | Qt | CCAGTGAGCAGAGTGACGAGGACTC GAGCTCAAGCTTTTTTTTTTTTTTTT | Pfu Ultra II Fusion HS DNA polymerase (Agilent Technologies, Santa Clara, CA, USA) | A2_1 | ATGCCTAAATTACCGCGTGGTCTGC |
| | | Qi | ACGAGGACTCGAGCTCAAGC | | A2_489 | TTTACTGCGCAGACTGCGTG |
| | | seq_3368 | TATCATGACGGTTCTGCCAG | | seq_3368 | TATCATGACGGTTCTGCCAG |
| | | | | | seq_3752 | AATCCCAGTCAACACGAGCG |
| | | | | | A153C_f | TTTATCGACTCTTCCGACACGCATC |
| Qi & beta 775 product | 1st strand cDNA with poly(T) and poly(A) | Qi | ACGAGGACTCGAGCTCAAGC | Pfu Ultra II Fusion HS DNA polymerase | beta_end | TTACGCCTCGTGTAGAGACGCAACC |
| | | beta_775 | CGGTTACACATTCGAGCTCG | | seq_512 | GGACTCACTATACGGTGACG |
| | | | | | beta_775 | CGGTTACACATTCGAGCTCG |
| | | | | | beta_273 | GTGATTACGGACCATACAAG |
| | | | | | beta_561 | CGGCAAGCACTACTATTCTG |
| A2_489 & seq_2281 product | 1st strand cDNA with poly(T) and poly(A) | A2_489 | TTTACTGCGCAGACTGCGTG | Phusion™ High-Fidelity DNA polymerase (New England Biolabs, Ipswich, MA, USA) | seq_2281 | ATACTTACCTGTCCCTGGCG |
| | | seq_2281 | ATACTTACCTGTCCCTGGCG | | seq_2870 | AGATCTAGTGTAGCGAACGG |
| | | | | | seq_3368 | TATCATGACGGTTCTGCCAG |
| | | | | | A2_489 | TTTACTGCGCAGACTGCGTG |
| | | | | | A2_2997 | ACAGACGTGACATCCGGCTC |
| | | | | | coat_2498 | CAGGCATATGCTGACGTGAC |
| coat_2498 & seq_1722 product | 1st strand cDNA with poly(T) and poly(A) | coat_2498 | CAGGCATATGCTGACGTGAC | Phusion™ High-Fidelity DNA polymerase | seq_1722 | GCAATTGTGCGCTGAGAGAG |
| | | seq_1722 | GCAATTGTGCGCTGAGAGAG | | seq_2281 | ATACTTACCTGTCCCTGGCG |
| | | | | | coat_2498 | CAGGCATATGCTGACGTGAC |
| | | | | | RT_1932 | TGCTTATTGCTCTCTTAGCG |
| seq_512 & RT_1932 product | 1st strand cDNA with poly(T) and poly(A) | seq_512 | GGACTCACTATACGGTGACG | Phusion™ High-Fidelity DNA polymerase | seq_512 | GGACTCACTATACGGTGACG |
| | | RT_1932 | TGCTTATTGCTCTCTTAGCG | | seq_1112 | TCTGTCTTACTGTTCTTAGG |
| | | | | | seq_1722 | GCAATTGTGCGCTGAGAGAG |
| | | | | | RT_1932 | TGCTTATTGCTCTCTTAGCG |
| | | | | | beta_815 | CGGTAGTGTTGTTACCTACGAG |
| | | | | | RT_plus_f1 | AAGCTGTTGCCTGGGAGAAG |
| seq_2234 & A153C_f product | 1st strand cDNA with poly(T) and poly(A) | seq_2234 | GGAGGCTCGTAAACCTCCTC | Phusion™ High-Fidelity DNA polymerase | seq_2234 | GGAGGCTCGTAAACCTCCTC |
| | | A153C_f | TTTATCGACTCTTCCGACACGCATC | | seq_2870 | AGATCTAGTGTAGCGAACGG |
| | | | | | seq_3368 | TATCATGACGGTTCTGCCAG |
| | | | | | A153C_f | TTTATCGACTCTTCCGACACGCATC |
| | | | | | A2_2997 | ACAGACGTGACATCCGGCTC |
| | | | | | coat_2498 | CAGGCATATGCTGACGTGAC |
| | | | | | A2_489 | TTTACTGCGCAGACTGCGTG |
| | | | | | A2_1158 | ACGAGCAATCTTCCGTTCGC |
